# Surgical Cases of Interest, Treated at Institute Hospital, Atlanta, Ga., May and June, 1864

**Published:** 1865-02

**Authors:** D. C. O'Keefe


### CONFEDERATE STATES MEDICAL & SURGICAL JOURNAL.

VOL. II. RICHMOND, FEBRUARY, 1865. No. 2.

### ORIGINAL COMMUNICATIONS.

ART. I.—*Surgical Cases of Interest, treated at Institute Hospital, Atlanta, Ga., May and June*, 1864. By Surgeon D. C. O'KEEFE.

Case 1.—Private W. F. Holmes, company "G," 4th Tennessee cavalry, age 24, occupation farmer, constitution good; wounded May 9th, admitted May 11th, 1864.

*History.*—Gun-shot wound requiring amputation of left thigh at its middle third, on the field, by flap method. May 20th.—Slight secondary hemorrhage from stump May 25th.—Stump gaping and bone protruding from inner angle; slight hemorrhage. June 1st.—Bone protruding half an inch. Patient placed under influence of chloroform, muscles dissected up from bone, thorough retraction used, and three-quarters of an inch removed with saw. The flaps were now completely adjusted with sutures, covering bone perfectly. Slight secondary hemorrhage occurred twice in one week after operation. June 30th.—Patient has had chronic diarrhœa since admission; is improving satisfactorily. Stump well cicatrized.

Case 2.—Jarrett Tucker, sergeant, 4th Tennessee cavalry, company "G," aged 40, occupation farmer, constitution good; wounded May 9th, admitted May 10th, 1864.

*History.*—Gun-shot wound requiring amputation of right thigh at junction of middle with upper third, on the field, by flap method. Stump, on admission, inclined to gape open, and on May 13th one inch of the bone was exposed. May 14th.—One and a quarter (1¼) inches of the bone was removed in the manner prescribed in the preceding case. Before resorting to operation in this case, an unsuccessful effort had been made to cover the bone by means of traction, with adhesive straps and a light wooden frame attached to extremity of stump. June 30th.—No untoward symptom since operation. Stump cicatrized; will be furloughed.

Case 3.—J. W. White, private, company "F," 39th Georgia, aged 40, occupation farmer, constitution feeble; wounded May 18th, admitted May 20th, 1864.

*History.*—Gun-shot wound, requiring amputation of left thigh at its middle third, on the field, by circular method. On admission, had high fever, with delirium and erysipelatous condition of stump, and tendency to diarrhœa. June 1st.—Changed from hospital building to tent, and improving a little; stump inclined to slough. June 10th.—Bone exposed, general condition better. June 12th.—One and a half (1½) inches of bone sawed off. June 20th.—Improved since last note; condition at present—tongue and abdomen tympanitic, with irritative fever. The stump sloughed a little, and despite the utmost care a troublesome bed-sore formed on the sacrum. June 29th.—Died of irritative fever.

*Remarks.*—This was a feeble old man, of small stature and delicate health. I cannot say that the secondary operation materially prejudiced his case.

Case 4.—W. B. Slaughter, private, company "D," 18th Tennessee, aged 20, occupation clerk, constitution good; wounded in battle of Chickamauga, September 19th, 1863, admitted February 20th, 1864.

*History.*—On admission patient was very feeble and anæmic, having been an inmate of hospital, Kingston, Georgia, from date of wound until his transfer to this hospital. The bone was exposed for half an inch, with a ledge of callus surrounding its extremity half an inch thick. The stump presented a conical shape, with great atrophy of the muscles of the thigh. March 15th.—Suffered a severe attack of inflammatory rheumatism, involving pericardium, which lasted one month. From this time his general condition improved a little; bone still exposed. June 15th.—Slight diarrhœa, still feeble and emaciated, stump has suppurated more or less freely from admission; end of bone black. Efforts were now made to remove the necrosed bone by daily twisting, which succeeded on the 25th, when about six inches of the necrosed extremity of the femur was removed.

*Remarks on the four preceding cases.*—There is no complication more frequent, perhaps, in hospital, than the exposed end of the femur from retraction and sloughing of the flaps. Whether this is owing to original deficiency of flap, or unavoidable retraction and sloughing of the same, is difficult to say; but it has seemed to the writer that the preference of field surgeons for the flap operation has some agency in the production of this most troublesome result. My notes furnish fifteen cases of this condition in nearly three years hospital practice. The first two were not interfered with, and they died from exhaustion and irritative fever; the last, Slaughter, is still under treatment. The necrosed bone having been removed on the 25th of June, by torsion, nine months after reception of wound; during this time he has been a great sufferer, and even now he is so much emaciated that his recovery is doubtful. In the remaining twelve cases from one-half an inch to two inches of the bone has been removed by dissecting up the muscles close to the bone, forcibly retracting the tissues with the retractor, and then using the saw. Ten (10) of the twelve (12) have recovered. But little blood is lost in this operation, especially if performed before much callus is thrown out around the extremity of the bone. If that has occurred, it will be difficult to make the dissection without considerable hemorrhage, which will compromise the safety of the patient. In a few cases the covering of the bone may be effected by means of traction with adhesive strips attached to a light frame made fast to the stump, or the extension may be made by means of a weight and roller over the foot of the bunk.

Case 5.—John Moore, sergeant, company "E," 38th Alabama, aged 24, occupation farmer, constitution good; wounded May 9th, admitted May 11th, 1864.

*History.*—Gun-shot wound, ball entering just over external cantpus of left eye, passing upward and backward, lodging under scalp near occipital protuberance, and fracturing skull in whole course of its track. On admission, examination showed that the skull was fractured and both tables depressed in an antero-posterior direction from point of entrance to point of extraction of ball. There was great tumefaction and discoloration of whole face and head; eye-lids closed firmly, which, when elevated, showed contraction of pupils. Patient completely unconscious and unable to move his limbs; pulse feeble and fifty, deglutition almost impossible, and the power of articulation entirely lost. Administered a scruple of calomel with great difficulty, which, on the following day, produced free purgation. May 12th.—Skin cool, pulse fifty, seemed to understand when spoken to. May 13th.—Could see a little out of right eye, pulse sixty; condition otherwise the same. May 14th and 15th.—Bowels acting freely from mercurials, pulse sixty and feeble, skin pleasant. May 16th.—General condition about the same, with constant gaping, brain substance sloughing out from anterior wound, pupils normal. May 17th.—General condition better, appetite improving, pulse ninety. May 18th and 19th.—No appetite, wounds discharging cerebral substance freely, soft tumor near posterior wound. May 26th.—Since last record he has had involuntary actions from bowels, pulse slow and feeble, (40), and was thought to be sinking, abscess near posterior wound opened and discharged freely, whole scalp and fractured portions of skull soft and yielding to the touch. From 26th to 30th condition somewhat better, brain still sloughing, nods his head when spoken to. June 1st.—Patient partially conscious and was transferred to a tent, can articulate a few words, several loose spiculæ of bone were removed, pulse regular. There is now well marked hemiphlegia of right side. June 30th.—Since last date he has gradually but steadily improved, and is entirely conscious, but articulates imperfectly; appetite good, sits up on his bed daily, and is rapidly gaining strength. Tumefaction has subsided, revealing depressed bone to the extent of six inches in length by four in width, wounds opened and suppurating.

*Remarks.*—This is a remarkable recovery from a severe and extensive injury of the skull. He is now convalescent, and will be sent home on furlough in a few days.

Case 6.—W. W. Gary, sergeant-major, 4th Georgia battalion, S. S., aged 18, occupation student, constitution good; wounded May 14th, admitted May 15th.

*History.*—Gun-shot wound, ball entering left groin at middle Puopart's ligament, injuring femoral artery and passing out near os coccygis, on same side. On admission, the wound of entrance was found enlarged, and the patient stated that the femoral artery had been tied at the field hospital, after a loss of a great deal of blood, no chloroform being used. Patient quite ensanguined, pulse 100, skin cool, very little swelling of limb, but complained of great pain in the left foot and leg. May 22d.—Since admission has had marked febrile paroxysms in the afternoon, pulse being 80 in the morning and 120 in the evening. Quinine was freely administered for this condition, with some apparent success; limb much swollen, with some discoloration, wounds healthy, but suppurating very freely. A distinct thrill perceptible to touch and auscultation over the whole of "scarpus triangle," but no swelling or tumor of the parts whatever. Constitutional symptoms favorable, though pulse was full and quick, with general arterial excitement. This condition lasted until the 27th of May, when hemorrhage occurred about one quart from the posterior wound. Much debility, ænema and fever followed this hemorrhage, with a chill on the 29th of May May 31st.—Another slight hemorrhage, about two (2) ounces, from the posterior wound, which occurred while having a motion from the bowels. June 1st.—Died. Asthenia.

*Remarks.*—The aneurismal thrill in this case was very perceptible, although there was no appreciable tumor. The hemorrhage evidently proceeded from some of the gluteal arteries, and hence no surgical interference was deemed advisable. The patient could give no reliable account of the operation performed on the field.

Case 7.—S. H Anderson, lieutenant, 34th Mississippi, company "B," aged 42, occupation druggist, constitution good; wounded May 14th, admitted May 22d, 1864.

*History.*—Gun-shot wound, ball entering near upper and posterior border of right os innominatum, passing obliquely downward through upper portion of rectum towards left thigh, lodging. On admission patient was suffering intense pain, with daily febrile exacerbations and constant fecal discharges from wound, which was suppurating very freely. Patient continued in this condition until May 30th, when diarrhœa set in, which continued, except when controlled by opiates and astringents, until his death, on June 5th, 1864.

Case 8—W. A. Tatum, private, company "B," 12th Tennessee, aged 25, occupation farmer, constitution good; wounded May 17th, admitted May 18th, 1864.

*History.*—Gun-shot wound from minie ball, entering at upper edge of forehead on the right side, fracturing the right parietal bone, causing depression of both tables of the skull. On admission, patient was fully conscious, free from all pain and able to walk about the ward, pulse normal; examination of wound showed that he had been trephined on the field, and the patient stated that the operation had been performed one hour after reception of injury, and that all spiculæ of bone had been removed. May 20th.—Pulse 70, slight head-ache. May 21st.—Doing well, symptoms improved. May 22d.—Severe pain in head and slight fever in afternoon. May 23d.—No fever during day, but great pain in right eye. May 24th.—Doing well; no pain. 25th.—Patient very dull, appetite gone, pulse feeble and intermittent. 26th.—Same condition, except slight rigor at 11 o'clock, A. M. 27th.—General condition unchanged, but great tumefaction of scalp and face. 28th and 29th —Pulse 65 and feeble, partially unconscious, feverish in afternoon. 30th and 31st.—No change except free sloughing of cerebral substance. June 1st.—Died.

*Remarks.*—This is the second gun-shot fracture of the skull, received from the recent action at the front, on which the trephine was used, and both have terminated fatally.

Case 9.—J. W. Chandler, private, company "C," 54th Georgia, aged 40, occupation farmer, constitution enfeebled by diarrhœa of fourteen months duration; wounded May 18th, admitted May 20th, 1864.

*History.*—Gun-shot wound requiring amputation of left thigh, middle third, by circular method. Stump sloughed from admission, and the diarrhœa could not be checked. Died June 2d, 1864.

Case 10.—J. N. Murphy, private, 37th Alabama, company "H," aged 27, occupation farmer, constitution moderate; wounded May 9th, admitted May 11th, 1864.

*History.*—Gun-shot wound from a spent ball entering right knee joint near internal condyle of femur. On admission, leg and thigh were greatly swollen, general condition very unfavorable. This condition continued until the 15th of May; wound at this time suppurating freely. From this time to June 1st the discharge from wound was very free, with considerable febrile action, night sweats and diarrhœa. There were also bronchitis and a chill. From June 1st to 10th thigh more swollen and very tender, diarrhœa controlled, condition otherwise unchanged. 10th to 20th.—Not much changed, free incision made for exit of pus just above wound, debility decided, with a troublesome cough. June 23.—Transferred to tent, another incision below wound, a black slough two (2) inches square formed, embracing wound. June 30th.—Condition somewhat more favorable, pulse 80 to 90, diarrhœa controlled, slough improving, but debility still great.

Case 11.—F. M. Robinson, sergeant, 2d Kentucky, company "I," aged 26, occupation farmer, constitution moderate; wounded May 14th, admitted May 17th, 1864.

*History.*—Shell wound over right parietal eminence, fracturing both tables of skull at that point to the extent of a half inch square, with depression of a portion of both tables to same extent. The skull was denuded over a surface as large as the palm of the hand. There was also a severe flesh wound about two inches square near inferior angle of left scapular. On admission, patient was conscious, but reports that he was unconscious for fourteen hours after receipt of injury. Great pain, with echymosis extending round right eye and over the face, pupils contracted, pulse slow and feeble, skin cool. From this time until 23d condition about the same, wound did not suppurate, suffered great pain over whole head, for which opiates were freely given. 25th.—Wound began to suppurate, and a soft fluctuating tumor formed, extending over left side of scalp and left eye. 30th.—Wound began to slough badly, both of scalp and back, some disposition to become gangrenous, also diarrhœa. June 1st.—Changed to a tent by himself, the object being fresh air and quietude. June 8th.—No change since last note, except convulsion on this day, followed by another on the 9th. June 10th.—Very cheerful, pulse regular, appetite good, wounds more healthy. June 11th.—Late at night had another convulsion. These convulsions were slight, not lasting over one to two minutes. June 12th.—The depressed plate of bone, half an inch square, was removed by sawing through a small triangular piece of the outer table with saw. The removal of this piece of bone gave easy access to the depressed portion, which, with several spiculæ, was removed with forceps and elevator. About a tea-spoonful of pus, with disorganized membranes and cerebral substance was removed at the same time; chloroform was used during the operation. 13th.—Very cheerful, no recurrence of convulsions. June 14th.—Another slight convulsion. June 30th.—Has kept steadily improving, wounds in a healthy, granulating condition, the large surface of denuded skull seems to be diminishing and covering over slowly. The pulse has not been over 90 at any time since his admission. There is every prospect of his recovery. There has been no paralysis.

Case 12.—S. H. Lary, private, Hiris' Battery, aged thirty-five, occupation farmer, constitution good; wounded May 26, admitted May 27, 1864.

*History.*—Gun-shot wound, ball entering on anterior aspect of forearm, two inches below external condyle, fracturing radius and ulna, and passing out three inches above on the inner side of the arm; also, gunshot wound, ball passing through adductor muscles of right thigh and making its exit above Poupart's ligament. On admission the patient stated that he had laid on the field from ten o'clock, A. M., till after dark on May 26, during which time he received the second wound. On the 27th he was moved in a wagon from the battlefield of New Hope Church to Marietta, a distance of about fifteen miles, and thence by rail to Atlanta, twenty miles. He was received in hospital twelve o'clock at night of the 27th. On examination of the injured limb, the whole of the inside of forearm was of a purplish dark color; no pulsation of radial artery. All the appearances of the limb indicated that it was rapidly passing into a state of inflammatory gangrene. Amputation by circular method was performed on morning of the 28th at junction of upper with middle third; chloroform was used; there was but little blood lost and re-action was speedy and complete; stump did well until June 1, when an offensive discharge with sloughing action commenced—general condition moderate. June 6—Slough began to separate; some indications of pyæmia now manifested themselves. June 7—Rigors and vomiting, evidently sinking. June 10—Died.

*Remarks.*—This man in all probability would have recovered, had he been operated on in the field. The same unhealthy action attacked the stump which involved the whole forearm before the operation. The severe wound in the groin also added much to the gravity of the case. He was isolated and kept in a small room by himself immediately after operation.

Case 13.—Virgil M. Lassiter, Thirty-Second Texas Cavalry, company "E," aged seventeen, occupation student, constitution good; wounded May 20, admitted May 21, 1864.

*History.*—Gunshot wound of middle third of right leg, badly fracturing both bones. On admission leg very much swollen, but no constitutional disturbance. This condition continued until June 1, when his system began to sympathize with the cruel injury, wounds enlarging but not suppurating healthily; pulse frequent. Constitutional disturbance became more marked from day to day, and on June 5th amputation of the limb was performed at the knee-joint, through the condyles. A long semilunar flap was obtained anteriorly, and a short one posteriorly, by transfixion, the patella being removed. Chloroform was used and re-action favorable. June 9—Considerable sloughing of posterior flap, with some constitutional disturbance. June 10—Slight secondary hemorrhage. From this date to 20th improved steadily; stump cicatrized beautifully. From 20th to 30th has suffered severely from paroxysmal fever, for which quinine has been freely used, and which is now abating. Prospect of recovery favorable.

Case 14.—W. J. Young, private, Sixteenth Louisiana, company "A," aged twenty-five, occupation farmer, constitution good; wounded May 8, admitted May 9, 1864.

*History.*—Gunshot wound, ball entering near middle of posterior border of scapulæ, two and a half inches from the spine, and passing upwards, made its exit anteriorly, fracturing the clavicle at its middle third. On admission patient stated that he had had profuse hemoptysis and dyspnea, with copious hemorrhage from wound, immediately after reception of injury. There was little or no constitutional disturbance. May 11—Cough and fever, which lasted several days, and for which anodynes with tr. aconite were given. May 15—Wounds suppurating freely, cough subsided. May 24—Posterior wound nearly healed; improved steadily from this time to June 4, when he was furloughed home in a convalescent condition.

*Remarks.*—This clearly seems to have been a penetrating wound of the chest, from which the recovery was very speedy and satisfactory.

Case 15.—M. V. Temples, sergeant, company "A," Second Arkansas regiment, aged twenty-four, occupation farmer, constitution enfeebled by previous wounds; wounded May 8, admitted May 9, 1864.

*History.*—Gunshot wound, ball entered just in front of left ear, passing upwards and forwards, made its exit just above the right eye. On admission patient was perfectly rational, with no constitutional disturbance; could see but little with right eye, and said that vision of left eye was lost from time of wound. May 11—A little wandering, pulse slow, has taken calomel frequently in purgative doses. May 16—Rational, with some appetite; 18th to 26th—Unconscious, but seemed at times to understand when spoken to very loudly; can still take food, but is very feeble and emaciated; involuntary fœcal discharges; wounds suppurating very freely. June 1—Transferred to a tent, improved a little, but died June 8, 1864, of asthenia.

Case 16.—J. F. Whitaker, private, company "K," Eighth Tennessee regiment, aged twenty-six, occupation farmer, constitution impaired by syphilitic taint; wounded May 17, admitted May 18, 1864.

*History.*—Gun-shot wound, ball entering just behind the right internal maleolus, and passing out at external maleolus, badly fracturing the lower extremity of fibula. On admission patient suffered great pain, with much constitutional disturbance; bowels alternately loose and constipated; wound of exit unhealthy. June 1—Foot became dark and cold, and wound assumed a gangrenous appearance, which increased until June 12, when amputation of leg was performed at junction of lower with middle third. The circular method was employed; chloroform was used, and the re-action was good. Examination of amputated limb revealed extensive fracture of lower extremity of fibula, and on opening into the ankle joint with disease of all its structures. Patient did well for four days after operation, when stump was attacked with unhealthy action. June 22—Sloughing of stump ceased and general condition improved. June 30—Condition, though critical, is still somewhat promising.

Case 17.—C. J. Collins, private, company "G," Fifty-Eighth Alabama regiment, aged forty-five, occupation farmer, constitution feeble; wounded May 11, admitted May 12.

*History.*—Shell wound of upper third of left leg, badly fracturing the tibia and requiring the removal of large spiculæ of bone on the field. On admission, general condition unfavorable; slight fever and tendency to diarrhœa, with sloughing of wound. May 18—Somewhat improved, granulations healthy. June 10—Diarrhœa, wound looks pale. June 15—Diarrhœa increased, anorexia and fever. These symptoms, with sloughing wound, continued to increase until June 25, when a marked improvement took place. June 30—Wound healthy, general condition promising.

Case 18.—T. D. White, private, company "C," Fifty-Eighth Alabama regiment, aged thirty, occupation farmer, constitution good; wounded May 14, admitted May 16, 1864.

*History.*—Gunshot wound of left shoulder-joint, fracturing bone composing same; also of right foot, fracturing first metatarsal bone. On admission had little or no constitutional symptoms, but on May 29 pyæmic symptoms set in and he died June 1, 1864.

Case 19.—E. Hufftickler, private, company "I," Thirtieth Mississippi regiment, aged thirty-eight, occupation farmer, constitution good; wounded May 15, admitted May 17, 1864.

*History.*—Gunshot wound of left arm, requiring amputation at the shoulder-joint on the field. On admission condition very unfavorable. May 18—Erysipelas involved the stump and gradually extended up the neck and over the face, and partially over the body. June 1—Erysipelas was in a great measure arrested by the free use of quinine and iron, but he died June 5, of cerebral symptoms.

Case 20—Joseph Step, private, company "I," Fortieth Georgia regiment, aged twenty-nine, occupation farmer, constitution good; wounded May 20, admitted May 27, 1864.

*History.*—Gunshot wound, ball entering just below the angle of right inferior maxilla and passing through the neck, made its exit about a corresponding point on the opposite side, same ball also fractured the left arm, causing amputation of same at its upper third on the field. On admission patient very weak from loss of blood; stump inclined to slough; could only swallow liquid nourishment; considerable constitutional disturbance. The symptoms increased in gravity until June 7, when secondary hemorrhage occurred from both wounds of the neck. Died June 8.

Case 21.—J. J. Shanks, sergeant, company "A," Seventeenth Alabama regiment, aged twenty-five, occupation farmer, constitution good; wounded May 20, admitted May 22, 1864.

*History.*—Extensive shell wound of outer side of left foot. On admission general condition good, wound inclined to slough. May 25—Wound cleaned out and granulations healthy. June 1—Pyæmic symptoms set in, of which he died June 8, 1864.

Case 22.—Thomas Gannon, private, company "D," Fifth Confederate, aged thirty, occupation boatman, constitution good; wounded May 16, admitted May 18.

*History.*—Gunshot wound, ball entering at right acromion process, fracturing the clavicle and passing through the muscles of the back of the neck, made its exit one inch below the lobe of left ear. On admission had high fever, with tendency to diarrhœa. May 25—A soft circumscribed abscess formed over middle of right clavicle, which on the 27th turned of a dark color and sloughed. May 31—A well-marked case of gangrene. June 7—Had a chill. June 9—Died.

Case 23.—J. Holden, sergeant, company "E," Second Arkansas regiment, aged twenty-two, occupation farmer, constitution good; wounded May 14, admitted May 16, 1864.

*History.*—Gunshot wound of left elbow joint, causing amputation (by flap method) of left arm at its middle third on the field. On admission patient prostrated from loss of blood and high fever; seemed to improve up to June 1, but still very feeble; stump cicatrizing well. June 3—Pyæmic symptoms developed themselves, of which he died June 18.

Case 24.—W. H. Young, private, company "E," Third Georgia Cavalry, aged eighteen, occupation student, constitution good; wounded June 11, admitted June 12, 1864.

*History.*—Gunshot wound, ball entering the abdomen on a line two inches above anterior superior spinous process of illium on the right side, ball lodging. On admission there was profound constitutional disturbance, nausea and vomiting, retention of urine, &c., which continued until his death, June 13, 1864.

*Treatment.*—Opium to a point short of narcotism. No autopsy could be obtained in this case.

Case 25.—G. W. Mosely, private, company "E," Second Missouri regiment, aged twenty-one, occupation clerk, constitution good; wounded June 1, admitted June 2, 1864.

*History.*—Gunshot wound, ball entering at posterior extremity of spine of right scapula, fracturing same and lodging; extracted at same point. On admission high fever, wound suppurating very freely, right arm paralyzed. June 18—Arm much swollen, wound very unhealthy. June 19—High fever, arm still much swollen from shoulder to hand. June 20—Sinking, arm in a gangrenous condition. June 21—Died.

Case 26.—S. D. Cannon, private, company "D," Second Alabama Cavalry, aged twenty-two, occupation merchant, constitution feeble; wounded May 18, admitted May 20.

*History.*—Gunshot wound, requiring amputation of left leg, just below the tubercle of tibia, on the field. On admission patient much depressed; fever, with retention of urine; stump greatly swollen, with a tendency to slough. His condition became worse from day to day until his death, on the 25th of June, from sloughing of stump and irritative fever.

*Remarks.*—This was a flap operation, most of the flap being obtained from the muscles of the calf.

Case 27.—Patrick Goode, corporal, company "G," Twenty-Sixth Tennessee regiment, aged twenty-two, occupation student, constitution enfeebled by dyspepsia; wounded May 15, admitted May 17, 1864.

*History.*—Gunshot wound, requiring amputation of right thigh at its lower third, on the field, by flap method. This patient suffered from admission with dyspeptic symptoms, daily nausea and vomiting, with a quick pulse which could not be controlled. June 17—Chill; stump remarkably healthy and cicatrizing rapidly. June 23—Two chills. June 24—Two chills. From this time he failed rapidly, and died of asthenia, June 27, 1864, with his stump healed.

Case 28.—W. H. Smith, private, 32d Mississippi, company "I," aged 19, occupation student, constitution good; wounded June 16th, admitted June 18th, 1864.

*History.*—Extensive shell wound over sacrum, exposing and fracturing the bone at the right sacro-illiac junction. This wound was 12 inches long by 8 wide. Had rigors for several days before death, which occurred June 28th, 1864.

Case 29.—J. H. Harrison, private, 42d Georgia, company "G," aged 25, occupation farmer, constitution good; wounded May 13th, admitted May 23d, 1864.

*History.*—Gun-shot wound, ball entering on inner side of tendo-achilis and passing directly forward beneath internal maleolus, partially involving ankle-joint. On admission, wound very painful; foot and ankle much swollen. May 30th, wound suppurating freely; healthy in appearance and less painful; a small loose angle of bone was exposed over internal maleolus, which was removed. June 9th, leg highly inflamed; swollen and painful from knee to ankle; suppurating from wound ceased; great constitutional disturbance; irrigation was used for inflamed limb; stimulants, quinine, iron, and nutritious diet, used internally. June 12th, inflammation increasing and threatening gangrene; pulse weak, scarcely perceptible. He lingered in this condition until death on the 16th.

Case 30.—Geo. E. Bowman, private, 9th Arkansas, company "E," aged 30, occupation farmer, constitution good; wounded June 18th, admitted June 22d, 1864.

*History.*—Gun-shot wound of chest, ball entering at a point two inches to the right of sternum and one inch below clavity, and passing in an oblique direction toward right shoulder, lodged. The same ball inflicted a severe wound on the hand which was in an elevated position. Hemoptysis followed immediately upon receipt of injury, and patient has coughed up blood more or less freely since. No trace of the ball can be found. June 30th.—Up to this date patient has suffered no untoward symptom, except a slight hacking cough, which yields readily to mild expectorants. He was furloughed home in a convalescent condition.

Case 31.—Capt. S. M. Rawlston, 6th Georgia cavalry, company "D," aged 38, occupation farmer, constitution good; wounded May 10th, admitted May 19th, 1864.

*History.*—Gun-shot wound, ball entering the left fore-arm anteriorly about two inches from elbow-joint, and passing obliquely upwards made its exit just above olecranon process, on the back of arm; great hemorrhage followed the injury. On admission arm very painful; wound suppurating freely; no radial pulsation at wrist. June 5th, patient has done well up to this time; had a chill, followed by fever; suppuration diminished; arm more painful. For this condition, quinine was used in forenoon; aperients and poultice to arm. June 13th, parts around elbow continues red, swollen and painful; free discharge of pus from incision. From this time forward patient steadily improved, and was furloughed June 29th.

Case 32.—Z. Castlebury, private, company "D," 23d Alabama, aged 19, occupation student, constitution feeble; wounded May 13th, admitted May 22d, 1864.

*History.*—Gun-shot wound upper third of right arm, badly fracturing and comminuting the same for several inches of its extent. Resection performed on the field on the day of injury, removing about six (6) inches of the humerus. On admission patient doing well, wound presenting a clean, suppurating surface. June 25th.—Patient doing well and furloughed this day.

Case 33.—Edward Dignin, private, company "B," 5th Confederate, aged 30, occupation farmer, constitution good; wounded May 15th, admitted May 17th, 1864.

*History.*—Gun-shot wound of left thigh, just above the knee, implicating joint and requiring amputation of the thigh at junction of lower with middle third, on the field, by flap method. June 30th.—Has progressed favorably, and is now doing well.

Case 34.—H. C. Coles, private, company "B," 4th Tennessee cavalry, aged 18, occupation student, constitution good; wounded May 19th, admitted May 20th, 1864.

*History.*—Gun-shot wound requiring amputation of right leg just below the knee, on the field, by circular method. June 30th.—Patient has progressed most satisfactorily, except a slight exposure of the spine of tibia, which, in a few days, became covered over. Furloughed this day.

Case 35.—R. E. Paxton, private, company "E," 9th Mississippi, aged 20, occupation farmer, constitution good; wounded May 15th, admitted May 19th, 1864.

*History.*—Gun-shot wound, ball passing through and comminuting both bones of the forearm. Amputation performed on the field same day, just below the elbow joint. May 29th.—No untoward symptom. Patient furloughed home to day.

Case 36.—J. F. Ragsdale, private, company "I," 2d Georgia cavalry, aged 25, occupation farmer, constitution good; wounded May 17th, admitted May 22d, 1864.

*History.*—Gun-shot wound, ball passing directly through the elbow joint. Amputation on the field at its upper third, leaving a short but beautiful stump. May 29th, patient furloughed this day, having never had a bad symptom.

Case 37.—W. J. Vandigriff, private, company "I," 18th Tennessee, aged 20, occupation farmer, constitution good; wounded May 27th, admitted May 28th, 1864.

*History.*—Gun-shot wound just above the elbow joint, producing a compound comminuted fracture of humerus and severely mangling the soft parts. June 30th, patient has done well; no bad symptoms; will be furloughed home in a few days.

Case 38.—R. J. Fleming, sergeant, company "D," 20th Tennessee, aged 25, occupation farmer, constitution good; wounded May 14th, admitted May 15th, 1864.

*History.*—Gun-shot wound requiring amputation of right leg just below the knee, on the field. May 25th, stump sloughing; dark offensive puritent discharge from stump; end of tibia exposed; no constitutional symptoms; appetite good and sleep refreshing. May 29th, flap re-adjusted by means of sutuers and adhesive strips. June 30th, stump cicatrized well.

Case 39.—Lieut. A. J Stuart, Hoskins' Mississippi Battery, aged 25, constitution good, occupation farmer; wounded May 19th, admitted May 21st.

*History.*—Cannon-shot wound requiring amputation of left thigh at middle third. On admission patient was doing well; stump well closed; free from pain and suffering very little. May 27th, occasional oozing of blood from stump. 29th, oozing still continues from same point within and behind the flap. May 30th, oozing of blood still continues more freely; sutures divided and flap separated to ascertain the cause of hemorrhage. An immense clot of dark blood filled up the cavity behind the flaps, created by the sloughing of the muscles; the hemorrhage was probably due to sloughing or degeneration of muscles; the structures under the clots were dark and pale and still disposed to ooze blood. The cutaneous portion of the flap was perfectly healthy and had partly emitted by the first intention over the sloughing mass of muscular tissue; the stump now thoroughly cleaned, cauterized with nitrate of silver, and dressed with lint saturated with chlorinated soda. This dressing was renewed for several days when the stump was found discharging healthy pus and all oozing of blood had ceased. June 30th, has progressed favorably from last record and now fairly convalescent.

Case 40.—Jno. R. Jones, private, company "C," 37th Mississippi, aged 20, occupation farmer, constitution good; wounded May 9th, admitted May 16th, 1864.

*History.*—Gun-shot wound, ball entering the arm about its middle third, and passing from behind forwards, fractured the humerus extensively and lodged just behind the skin in front. The ball and several fragments of bone were removed on the field. On admission, arm much swollen and exceedingly painful; suppurating profusely and pus burrowing towards fore-arm. May 22, spiculæ of bone removed; wound has healthy appearance but suppurating profusely; splints and roller bandage applied to arm, the latter commencing at the hand. From this time forward the patient slowly improved until June 16th, when he was furloughed home in a promising condition.

Case 41.—Lieut. J. F. Stewart, company "F," 33d Alabama, aged 30, occupation farmer, constitution good; wounded May 28th, admitted 29th, 1864.

*History.*—The following account of the injury and treatment, was furnished the patient by Surgeon E. A. Flewellen, at Marietta: "Gun-shot comminuted fracture of the radius and ulna," ball and attached fragments of bone removed; sharp points of extremiets also removed. It is not believed that the elbow joint is laid open, but there is some doubt whether the ulna may or may not be fissured in the joint. On admission patient did well. In a day or two afterwards suppuration set in and has continued since in the greatest profusion. Pus of a dark grumous character flowed from the wounds in the greatest quantity, and also from punctures made in different parts of the arm. June 30th, patient has continued to grow worse and to all appearances is now rapidly sinking from exhausting suppuration.

Case 42.—N. J. Duke, private, Johnson's Battery, Artillery, aged 25, occupation farmer, constitution good; wounded May 13th, admitted May 21st, 1864.

*History.*—Gun-shot wound, ball entering the right arm on the outer side about two inches below the shoulder joint, passing directly towards the chest and lodging. On admission patient doing well; has had hemoptysis since reception of injury; no constitutional symptoms; wound healthy, with little or no suppuration. May 22d, considerable pain in right side; expectorates blood; no suppuration from wound; poultice to wound; opium and ipecac to relieve cough. May 27th, bloody expectoration still continues freely, with pain in side; slight fever; tincture of aconite and tincture of opium prescribed, with pepper poultice to side. June 1st, continues to expectorate bloody mucus, which has the appearance of a clot of blood which had been subjected for a time to the action of water; seized in afternoon with sudden very acute pain in side corresponding with lower lobe of right lung; cough or deep inspiration causes intense pain; pulse excited; skin hot; expectoration more bloody; ordered calomel 20 grains, Dover's powders 10 grains, to be given at once. June 2d, pain in side less intense; cough still very troublesome, but less painful; expectoration much augmented; bowels moved freely this morning; resume the aconite mixture. June 8th, patient doing well; wound rapidly healing; fractured humerus uniting; pulmonary trouble very slight, although he continues to cough up bloody mucus occasionally. June 13th, bloody expectoration and cough have ceased. June 30th, doing well; furloughed.

Case 43.—A. Oliver, private; company "G," 30th Alabama regiment, aged 45, occupation farmer, constitution robust; wounded May 27th, admitted June 3d, 1864.

*History.*—Gun-shot wound, ball entering one-and-a-half inches to the left of the ensiform cartilage and passing obliquely downwards over the cartilages of the last two ribs, lodged. Patient thinks the ball has lodged in the pelvic cavity, from a peculiar sensation as though a loose foreign body were present in that region, but examination per rectum failed to discover it. On admission wound very painful; great tenderness upon pressure over epigastric and left hypochondriac regions; some febrile excitement internally, and urinal functions normal. June 5th, great irritability of stomach; wounds suppurating and looking well; bowels loose and attended with great tenesmus. From this time forward patient gradually improved, until the 15th of June, when he was seized with a chill, followed by fever and sloughing phagedæna of wound. Patient was then removed to a tent. June 30th, sloughing action ceased; wound looks healthy; patient rapidly recovering.

Case 44.—Jas. Cotton, private, company "I," 33d Alabama regiment, aged 25, occupation farmer, constitution good; wounded May 27th, admitted June 3d, 1864.

*History.*—Gun-shot wound, ball entering the back about three inches to the right of the spine, opposite the fifteenth dorsal vertebra and passing in an oblique direction towards the spinal column, lodged. On admission appearence of wound healthy, slightly suppurating; complete paralysis of lower extremities with loss of sensation; also, paralysis of bowels and bladder. Two days after admission erysipelas of a very diffused character was developed, which continued to spread rapidly until it almost enveloped the whole body. June 9th, died.

Case 45.—James M. Smith, private company "D," 33d Alabama, aged 20, occupation farmer, constitution good; wounded May 27th, admitted June 3d, 1864.

*History.*—Gun-shot wound, ball entering the thigh in the middle of its upper third anterior aspect, a little to the outer side of median line, and passing directly backwards made its exit on the posterior lateral aspect of the limb, fracturing the femur in its course. On admission wound painful, but little suppuration; limb very much swollen as far as the knee; removed splints applied by field Surgeon and applied cold water dressing to the entire thigh. June 13th, doing well except considerable curvature of the thigh outwards, with undue shortening of the limb. After extension and straightening of the limb, a light, single, long splint with foot board, was applied to its outer side, extending from the foot to a point above the hip, and confined to the body. An opening was cut out to permit the dressing of the wound. By this means the length of the limb was increased about three inches and the deformity overcome. June 30th, has steadily improved since last note and is now doing well. There is a good prospect of recovery in this case.

Case 46.—W. H. Hagy, private, company "F," 58th North Carolina, aged 20, occupation farmer, constitution feeble; wounded May 9th, admitted May 19th.

*History.*—Gun-shot wound, ball entering the thigh at junction with middle and upper third, and passing directly from before, backwards, produced a comminuted fracture of the femur. On admission wound painful and suppurating freely; patient feeble and troubled with severe cough and diarrhœa. June 13th, patient's general condition no better. Several fragments of detached bone can be distinctly felt beneath the skin near the wound of entrance. Patient was put under the influence of chloroform and a free incision was made on the anterior surface of the thigh, and about one dozen of large fragments of bone which were detached, were removed. From this time forward the patient grew gradually worse and more debilitated, (as he had done before the operation,) until worn down from irritation, diarrhœa, and the excessive drain of suppuration. He died June 29th, 1864.

Case 47.—Lieut. Jas. Jackson, company "F," 48th Tennesse regiment, aged 27, occupation farmer, constitution moderate; wounded May 13th, admitted May 18th, 1864.

*History.*—Gun-shot wound, ball entering at the middle third of thigh anteriorly, produced a very severe fracture of the femur. On admission wound very painful; beginning to suppurate; general condition feeble; placed the limb in a straight fracture box and applied cold water dressing; ordered stimulants and nutritious diet. May 24th, wound suppurating profusely; odor very offensive; great debility; wound swollen and painful even below the knee. In this way the patient grew worse from day to day, suffering from the great drain of suppuration and the irritation of high febrile excitement every afternoon. Quinine, tonics, stimulants, &c., were administered freely, but to no purpose. The patient died June 9th—æsthenic.

Case 48.—Berrien Summerlin, private, company "G," 47th Georgia, aged 35, occupation farmer, constitution ordinary; wounded May 13th, admitted May 18th, 1864.

*History.*—Gun-shot wound, ball entering the back about two-and-a-half inches to the left, and directly opposite the middle dorsal vertebræ, and passing obliquely forwards, made its exit between the fourth and fifth floating ribs, about four inches to the left of the median line. On admission wound of entrance clean and healthy and suppurating kindly. Wound of exit closed, and has the appearance of a hard tumor; general condition good; free from cough, fever and pain. June 4th, patient continued to improve until to-day, when he was seized with a troublesome hacking cough, each paroxysm of which caused a jet of dark sanious and very offensive fluid to flow from wound of entrance; no pain, and but little constitutional disturbance; ordered ipecac and opium for cough; nutritious diet, &c. June 5th, dark sanious matter continues to flow from wound of entrance in forcible jets during each expiratory efforts of coughing; during inspiration air rushes into the wound with a hissing sound; cough continues troublesome, but without expectoration, except white mucus; auscultation reveals, both amphoric respiration and metallic tinkling; patient has considerable fever; general treatment continued. In this way the patient continued, without much change of interest, until June 13th, when the dark sanious discharge was substituted by healthy pus in moderate quantities. Shortly after this an abscess formed around the wound of exit and discharged freely of pus. June 30th, patient doing well, with a good prospect of recovery.

Case 49.—B. F. Ragsdale, private, company "A," 25th Arkansas, aged 25, occupation farmer, constitution feeble; wounded May 18th, admitted May 22d, 1864.

*History.*—Gun-shot wound, ball entering the side about midway the cartilage of the last floating rib, on right side, and passing obliquely backwards, made its exit between the spinal processes of second and third lumbar vertebræ; no paralysis followed the injury. On admission patient was doing well; aperture of exit discharging pus mixed with blood; general condition feeble.

*Treatment.*—Cold water dressing, stimulants and opium. June 1st, no change since last note. He was now seized with severe rigors every few hours, followed by high febrile excitement; wound discharging freely of dark grumous pus, mixed with a cheese-like matter resembling grains of sand floating in a fluid; tongue dry; pulse quick and feeble; trembling of limbs and delirium. From this time until the 4th, the symptoms of pyæmia became fully developed, with great prostration of all the vital forces and death.

Case 50.—E. H. Meyers, company "C," 19th regiment Alabama volunteers, aged 22, constitution good, occupation farmer; wounded May 20th admitted May 21st, 1864.

*History.*—Gun-shot wound, ball passing from before, backwards, and lodging just under the skin opposite and to the inner side of the head of the humerus, from which it was removed on the field. On admission the wound looked clean and healthy and discharged pus quite freely. May 25th, patient not doing well; wound has healthy appearance, but his general condition is unfavorable; patient complains of great pain in axilla of sound side, in which situation the skin was observed to be red and swollen; rigors, fever, tremors, jaundice, delirium, and great prostration of the vital powers, rapidly supervened, and he died of pyæmia poisoning, May 28th, 1864.

Case 51.—M. S. Sinclair, private, company "C," 36th Mississippi, aged 22, constitution good, occupation farmer; wounded May 27th, admitted May 29th, 1864.

*History.*—Gun-shot wound, ball entering right side about midway the sixth rib, and passing obliquely backwards, made its exit at the lower angle of the scapula. On admission patient very feeble from the loss of blood; wound healthy; ribs very little injured; great hemorrhage followed receipt of wound, but no hemoptysis. June 1st, patient arose from his bed without calling for assistance and immediately fell in a state of syncope, from which he could not be restored. He died in eight hours after the accident.

*Remarks*.—Profuse hemorrhage had probably occured from the wound and discharged internally, although there was no external evidence of it.

Case 52.—P. F. Bryant, private, company "G," 28th Tennessee, aged 25, constitution feeble, occupation farmer; wounded June 18th, admitted June 19th, 1864.

*History.*—The following notes were furnished by the patient: "Resection of the metarcarpal bone of the index finger and trapezium of right hand; fracture of right femur; ball lodged; health of patient bad when wounded."—C. B. Wilson, Surgeon 28th Tennessee. "This case was examined here (Marietta,) this morning. The femur is fractured near the trochanter, but not much comminuted. A portion of the ball was removed from medullary canal; more of it still remains, (as indicated by Nelaton's probe,) but could not be extracted. It is probable that a portion of the ball passed through the bone and lodged on inner side of thigh."—E. A. Fluellen, Surgeon, &c. On admission patient was very feeble, and continued to grow worse rapidly until his death on the following night.

Case 53.—Cyrus L. Nabors, sergeant company "F," 2d Arkansas, aged 29, constitution good, occupation farmer; wounded May 19th, admitted May 20th, 1864.

*History.*—Gun-shot wound by minie ball, which entered posteriorly two-and-a-half inches to the left of the spinal column, opposite the body of the fifth dorsal vertebræ, and passing obliquely forward, made its exit between the third and fourth ribs, at a point three inches to the left of the sternum. Alarming hemorrhage and hemoptysis followed the receipt of the injury. On admission patient was greatly debilitated from hemorrhage; there was neither cough, constitutional disturbance, nor hemoptysis; no pulse could be felt at the wrist in the radial artery of the effected side, nor in the brachial as far as the axilla. From admission this patient continued to do well without a single bad symptom. The arm on the wounded side was considerably atrophied and somewhat paralysed, due, doubtless, to the cutting off of the supply of blood and nervous influence. June 30th, doing well; furloughed home.

*Remarks.*—This case presents two points of deep interest: The first is that of an unquestionable wound through the lung without any symptom indicating that injury, except the original hemoptysis. The second was the undoubted severing of the left subclavian artery, as indicated by the absence of pulsation in the radial or brachial artery, which was carefully and frequently sought for, and of the absence of which there could have been no mistake.

Case 54.—A. J. Chandler, lieutenant, company "G," 40th Georgia, aged 25, constitution moderate, occupation farmer; wounded May 26th, admitted May 29th, 1864.

*History.*—Gun-shot wound, ball entering the right leg at its middle third, and passing directly through from before, backwards, severely fracturing the tibia. On admission leg much swollen and very painful; wound discharging thin bloody fluid; surface around the wound covered with vesicles filled with serum and blood; general condition good.

*Treatment.*—Fracture box, aperients, cold water, and opiums. June 10th, very little change, except the wound has suppurated freely until now. Patient suffers from intermittent fever, to which he was subject before being wounded. This complication lasted several days in spite of all remedies for its relief. The wound in the mean time suppurated freely and the limb was very much swollen. A large ulcer, also, made its appearance on the under surface of the leg, which manifested a great disposition to slough and spread until it assumed a serious aspect. June 21st, the patient continuing to grow worse; amputation was performed at the knee joint through the condyles, leaving the patella. Patient sustained the operation well and for a few days progressed favorably. Sloughing of the stump has since set up, and at this time, (June 30th,) seriously complicates the case.

Case 55.—W. F. Phillips, private, 37th Georgia, company "F," aged 21, constitution good, occupation clerk; wounded June 19th, admitted June 20th, 1864.

*History.*—Shell wound, a fragment of same entering at the fifth metacarpal bone of the right hand, fracturing the same, as also the fourth, and passing out through the palm of the hand between the first and second metacarpal bones. On admission the hand was greatly tumified, with considerable constitutional disturbance. June 26th, patient had secondary hemorrhage about one pint; wound unhealthy and gangrene threatening hand and fore-arm. The arm was now amputated at its middle third by the circular method. June 30th, doing well.

